# Evaluation of Performance Parameters of the Disposable Flexible Ureterorenoscope (LITHOVUE) in Patients with Renal Stones: A Prospective, Observational, Single-arm, Multicenter Study

**DOI:** 10.1038/s41598-018-28247-7

**Published:** 2018-06-28

**Authors:** Sung Yong Cho, Joo Yong Lee, Dong Gil Shin, Ill Young Seo, Sangjun Yoo, Hyung Keun Park

**Affiliations:** 1 Department of Urology, SMG-SNU Boramae Medical Center, Seoul, Korea; 2 Department of Urology, Severance Hospital, Urological Science Institute, Yonsei University College of Medicine, Seoul, Korea; 3 Department of Urology, Pusan National University School of Medicine, Busan, Korea; 4 Department of Urology, Wonkwang University School of Medicine and Hospital, 344-2 Sinyong-dong, Iksan, 570-711 Korea; 5 Department of Urology, Asan Medical Center, College of Medicine, University of Ulsan, Seoul, Korea

## Abstract

We investigated performance parameters of disposable flexible ureterorenoscopy (LITHOVUE) in patients with renal stones in a prospective, multicenter, observational study. Sixty two patients who underwent ureterorenoscopic surgery by LITHOVUE were included. Surgeons using a numerical scale, evaluated the maneuverability of the scopes and general perceptions of visibility related to the monitor and irrigation systems. General pain and fatigue scores were evaluated and compared to existing scopes. Mean patient age was 57.3 years and stone size was 15.4 mm. Clinical success of overall stone removal was 82.3%. Complications occurred in 4 cases of the Clavien classification grade I in a single case and II in 3 cases. Comparative parameters of maneuverability, perception of the monitor system and perception of the irrigation channel were 2.5, 2.5, and 3.0, respectively. The most favorable evaluation of physical strain was about ‘shoulder fatigue’ and ‘hand fatigue’. Unfavorable evaluations were recorded for ‘wrist stiffness’ and ‘thumb fatigue’. Maximal deflection angles of 270° were preserved in 53 cases (85.5%). No pre-stenting procedure and the longer operative time were significant predictors of poorly-preserved deflection angles <270°. LITHOVUE showed good maneuverability and perception scores for the monitor and irrigation systems. LITHOVUE received favorable evaluations on pain and fatigue scores.

## Introduction

With advances in technology, flexible ureteroscopy is the most rapidly growing intervention for active removal of urolithiasis. One of the most important hurdles for the use flexible ureteroscopes is cost-effectiveness^1,2^. Because of scope fragility, surgeons need to improve the high prices in buying and maintaining scopes. Maintenance fee for scope sterilization is another issue, and one which is particularly important for patients with microorganisms resistant to multiple antibiotics. However, the cost analyses can have limitations and they cannot be easily applied to the situation of other countries because there are significant differences in surgeons’ experience, surgical techniques, the types of flexible ureterorenoscopes, national reimbursement system, the time of evaluation of the current investigation, the price of scopes and maintenance, etc.

Recently, disposable ureteroscopes have emerged as a solution for the above-mentioned problems and a novel, single-use, flexible digital ureterorenoscope (LITHOVUE) was adopted in Korea^3^. LITHOVUE is the first disposable digital flexible ureterorenoscope in the world. Previous investigations showed good quality of the operative field of view and good maneuverability. However, these were based upon animal data or data acquired in a single center^4,5^. There has not been sufficient data gathered on human use^3^.

LITHOVUE is much lighter than previous reusable scopes^6^. It uses complementary metal oxide semiconductor (CMOS) digital technology and the scope is connected to its own portable monitor system. These characteristics make a difference in daily practice and in the selection of scope for stone surgery. However, there is a lack of clinical data regarding maneuverability, perceptions of the monitor system, perceptions of the irrigation channel, and ergonomic parameters such as pain or fatigue in shoulder, hand, wrist and thumb. Furthermore, many investigators with wide experience of stone surgery are in agreement about presence of ‘Asian ureters’ and narrow pelvic cavity^7^, referring to the narrow diameters of ureters in Asian populations. Although it has been poorly investigated, this necessitates that a clinical study be performed to determine the clinical value of this new disposable ureteroscope in the Asian population.

Therefore, the aim of this study was to evaluate the performance parameters such as maneuverability and perception of ergonomic parameters of the disposable flexible ureterorenoscope (LITHOVUE) in patients with renal stones in a prospective, multicenter, observational study, performed for the first time in Asia, Middle-East, and African areas.

## Methods

### Patients and study design

Consecutive patients who underwent active removal of stones in the kidneys and upper ureters by LITHOVUE were included in the study. All cases were primary ureteroscopic procedures. Informed consent was obtained from all participants for use of their medical records. The institutional review board of SMG-SNU Boramae Medical Hospital approved the observational study design and use of patient’s medical database (approval number: 16-2013-103). This study has been performed in accordance with the Declaration of Helsinki.

The surgeons at each institution were experts who had performed 500 to 1,500 cases of flexible ureteroscopic surgery. Eligible patients aged >20 years with planned unilateral ureterorenoscopic stone surgery were included and the inclusion criteria for surgical removal were based on the European guidelines for urolithiasis. Patients with complete ureteral obstruction, ureteral stricture, ongoing urinary tract infections, active bleeding, and anatomical deformities of horseshoe kidneys and ureteropelvic junctional obstruction were excluded. The authors anticipated the final number of patients in the analysis would be 50 and therefore the total number of patients enrolled was 62 in anticipation of a 20% drop-out rate. Sixteen patients were enrolled at each of two institutions (surgeons, Cho SY and Park HK) and 10 patients were enrolled at each of three institutions (surgeons, Lee JY, Shin DG, and Seo IY).

### Surgical methods

Flexible ureteroscopic procedures were followed by the same methods in the previous investigations^7^. All patients underwent cystoscopic or ureteroscopic examinations in the dorsal lithotomy position. A 0.035-mm Terumo guidewire (Terumo, Japan) was inserted into the ureteral orifice. The Terumo guidewire was changed into a Superstiff guidewire (Boston Scientific Corporation, Miami, FL, USA) using an open-ended 5-Fr ureteral catheter or a Dual lumen catheter (Boston Scientific Corporation). A safety guidewire using another Terumo guidewire was basically introduced. An 11/13 or 12/14 ureteral access sheath (Boston Scientific Corporation or Olympus Corporation, Tokyo, Japan) was introduced into the level of ureteropelvic junction or 2 to 3 cm below the targeted upper ureter stones. If the ureteral access sheath could not be introduced due to the ureteral narrowing, the sliding technique over the guidewire was performed: (i) the Superstiff guidewire was changed into a Terumo guidewire; (ii) a flexible ureteroscope was inserted into the renal pelvis though the Terumo guidewire. The LITHOVUE was introduced into the renal pelvis though the ureteral access sheath.

A 365- or 200- µm laser fiber was used to perform dusting or fragmentation techniques. Holmium laser was set to 10 to 30 W. For stone fragmentation, the energy was set to 1.0 to 2.0J with 5–10 Hz. For dusting or pop-dusting technique, the energy was set to 0.3 to 0.8J with 15–30 Hz. An endoscopic irrigation system (Stryker, Michigan, USA) or a manual irrigation system (syringe technique or HiLine, Coloplast, Denmark) was used to maintain an appropriate low intrarenal pressure to guarantee enough space to break the stones and to avoid intrarenal backward flow. The fragmented stones were removed by a zero-tipped nitinol stone basket (Boston Scientific Corporation). A double-J stent was routinely inserted and removed within postoperative one week. However, the double-J stent was removed at 2 to 4 weeks after surgery when there was ureteral narrowing.

### Clinical parameters and postoperative management

Patients’ demographics including age, gender, metabolic components, laboratory results of hepatic and renal function, and hemoglobin level were evaluated. Stone parameters included stone burden, location of stones, radiopacity, number of insertion of devices, types of devices used, stone composition, and remnant stone profiles. Hounsfield unit (HU) was used to measure stone density and stone volume was calculated by the formula of 0.523 × length × width × height. Non-contrast computed tomography (CT) scans were acquired preoperatively and follow-up images of plain KUB X-ray or CT scans were obtained postoperatively within 30 days to assess the presence of residual stones. Stone-free status was defined as ‘success’ when there was no stone fragment and ‘clinical success’ when there was no remnant stone >2 mm on the follow-up images. None of the patients got medication which can affect the stone-free rate. The stone distribution was evaluated by the Seoul National University Renal Stone Complexity (S-ReSC) scores. It is based on the 9 spaces involved in the renal collecting system^8,9^. The operative time was measured from the time of insertion of cystoscopes or ureteroscopes into the urethra to the indwelling urethral Foley catheter.

Surgeons evaluated perspectives on the maneuverability of the scopes, general perceptions of visibility related to the monitor system during surgery and visibility related to irrigation during the stone dusting or fragmentation. General pain and fatigue scores were evaluated after surgeons used the LITHOVUE and compared it to existing scopes^10^. Evaluated parameters included ‘shoulder stiffness’, ‘wrist stiffness’, ‘thumb fatigue’, and ‘hand fatigue’. The comparative scores of 1 (much better) and 2 (better) would mean a superior assessment of the LITHOVUE over existing flexible scopes in each institution. Scores of 4 (poorer) and 5 (much poorer) would mean a poorer judgement for LITHOVUE compared to existing flexible scopes.

All parameters were represented as the mean value ± standard deviation or number of cases. Independent t-test or Mann-Whitney U test was performed to compare the results between two groups. Categorical variables were tested by the Chi-square and Fisher’s exact test. The one-way analysis of variance (ANOVA) and Krukal-Wallis test were used to determine whether there are any statistically significant differences between the means of comparative parameters of the maneuverability, perception of the monitor system, and perception of irrigation system. Statistical significance was considered at *P* < 0.05. Statistical analyses were performed by IBM SPSS Statistics version 20 (IBM, Chicago, IL, USA) and R version 3.0.1 (http://www.r-project.org).

### Data availability statements

The datasets generated during and/or analyzed during the current study are available from the corresponding author on reasonable request.

## Results

### Patients and stone characteristics

Patient demographics are as shown in Table 1. A total of 62 patients were enrolled and no patient dropped out during the study period. The mean age was 57.3 ± 13.9 years and the stone size was 15.4 ± 5.4 mm. There were no significant differences in stone size, operative time and stone volume among surgeons.

**Table 1:** Patients and stone characteristics.

Variables	Variables
Mean ± SD or no. of patients (%)	
Patient characteristics	
No. of patients	62
Age (y)	57.3 (±13.9)
Male: female	38 (61.3%): 24 (38.7%)
Body mass index (kg/m^2^)	26.0 (±4.6)
Creatinine	
Preoperative (mg/dl)	0.9 (±0.3)
Change within 1 months postoperative (mg/dl)	0.9 (±0.3)
Estimated GFR	
Preoperative (mL/min/1.73 m^2^)	83.3 (±20.4)
Change within 1 months postoperative (mL/min/1.73 m^2^)	−1.0 (±1.5)
Hemoglobin	
Preoperative (mg/dl)	13.7 (±1.8)
Immediate postoperative minus preoperative (mg/dl)	−0.9 (±1.5)
Diabetes/hypertension	13 (21.0%)/26 (41.9%)
Stone characteristics	
Previous ESWL history	13 (21.0%)
Previous URS history	3 (4.8%)
Presence of hydronephrosis without obstruction	27 (43.5%)
Infundibulo-pelvic angle (°)	53.1 (±18.2)
Houns-field unit	761.9 (±348.6)
Diverticular stone	1 (1.6%)
Pre-stenting	22 (35.5%)
Stone laterality: Right/Left	29 (46.8%)/33 (53.2%)
Radiopaque/radiolucent	51 (82.3%)/11 (17.7%)
S-ReSC scores: 1–2/3–4/ ≥ 5	49 (79.0%)/9 (14.5%)/4 (6.4%)
Maximal stone size (mm)	15.4 (±5.4)
Total stone volume (mm^3^)	1376.2 (±1433.2)
Number of stones	2.0 (±16)
Main stone composition	
Calcium oxalate monohydrate	48 (77.4%)
Uric acid	11 (17.7%)
Carbonate apatite	3 (4.8%)

### Surgical outcomes

The clinical success of overall stone free rate was 82.3% and complete success rate was 64.5% as shown in Table 2. Maximal diameters of remnant stones ranged from 2 mm to 8 mm.

**Table 2:** Peri-operative characteristics.

Variables	Variables
Operative characteristics	
Safety guidewire	59 (95.2%)
Size of ureteral access sheath: 11–13Fr/12–14Fr	35 (56.5%)/27 (43.5%)
Operative time (min)	68.9 (±38.0)
Estimated blood loss (ml)	8.2 (±15.6)
Discharge (day)	1.6 **(**±0.6)
Removal of ureteral Double-J stent (day)	12.8 (±6.7)
Comparative parameters of LITHOVUE	
Maneuverability	2.5 (±0.9)
Perception of the monitor system	2.5 (±1.0)
Perception of the irrigation system	3.0 (±0.8)
Surgical success	
Complete/Clinical (no stone >2 mm)	40 (64.5%)/51 (82.3%)
Complication	4 (6.4%)
Clavien grade I: intractable pain and hematuria	1 (1.6%)
Clavien grade II: fever	3 (4.8%)

Complications occurred in 4 cases of the Clavien classification grade I in a single case (persistent hematuria and pain for >2 weeks) and II in 3 cases (fever). These complications were completely resolved with medications of analgesics or antibiotics, 4 weeks postoperatively. A single case was converted to combined procedure with percutaneous nephrolithotomy for removal of a diverticular stone in a modified Valvidia-Galdacko position.

### Performance of LITHOVUE

When LITHOVUE was compared to the existing scopes in each institution, assessments of maneuverability, perceptions of the monitor system and perceptions of the irrigation channel, were 2.5 (better to no difference), 2.5, and 3.0 (no difference), respectively (Table 3). Good evaluations for LITHOVUE were found in 25 cases (40.4%) of maneuverability, 25 cases (40.4%) of the monitor system and 15 cases (24.2%) of the irrigation system. Poor evaluation of LITHOVUE was found in 5 cases (8.1%) of maneuverability, 5 cases (8.1%) of the monitor system and 15 cases (24.2%) of the irrigation system. LITHOVUE received generally favorable evaluations on pain and fatigue scores from all surgeons. The most favorable evaluation related to ‘shoulder fatigue’ and ‘hand fatigue’. However, unfavorable evaluations were given for ‘wrist stiffness’ and ‘thumb fatigue’.

**Table 3:** Pain and fatigue scores after use of LITHOVUE compared to preexisting scopes.

No. of surgeons who evaluated the score	Very good	Good	No difference	Poor	Very poor
Shoulder stiffness	4	1	0	0	0
Wrist stiffness	3	1	0	1	0
Thumb fatigue	2	0	1	1	0
Hand fatigue	2	2	1	0	0
Other area (Please describe _________)	0	0	0	0	0

Maximal deflection angle of 270 degrees was preserved in 53 cases (85.5%). Impaired deflection angles at the end of surgeries were 260 degrees (1 case), 240 degrees (3 cases), 230 degrees (1 case), and 180 cases (4 cases). The logistic regression analysis showed that the well-preserved group of 270 degrees and the poorly-preserved group of <270 degrees showed significant differences in the rate of pre-stenting (95.0% vs 68.2%, OR = 9.19, 95% CI 1.11–76.2, *P* = 0.040) and the operative time (64.2 min vs 97.0 min, OR = 1.1, 95% CI 1.001–1.146, *P* = 0.041). Patient characteristics, maximal stone size, stone volume or the location of stone did not make significant differences of preservation of deflection angles during surgery.

The malfunction rate of the LITHOVUE during our study was 2 of 62 cases (3.2%). One was a connection error between the scope and the monitor. The other was due to distorted lateral direction of the flection.

## Discussion

There has been a paucity of clinical data about LITHOVUE. This is the first study to evaluate the performance parameters such as maneuverability and perception of ergonomic parameters of LITHOVUE in patients with renal stones in a prospective, multicenter, observational setting. Because LITHOVUE is a disposable device, it would seem to be appropriate for beginners in stone surgery considering scope fragility. Additionally, perceptions of ergonomic parameters need to be evaluated by surgeons with experience of multiple reusable flexible ureteroscopes.

### Maneuverability

**Figure 1:**
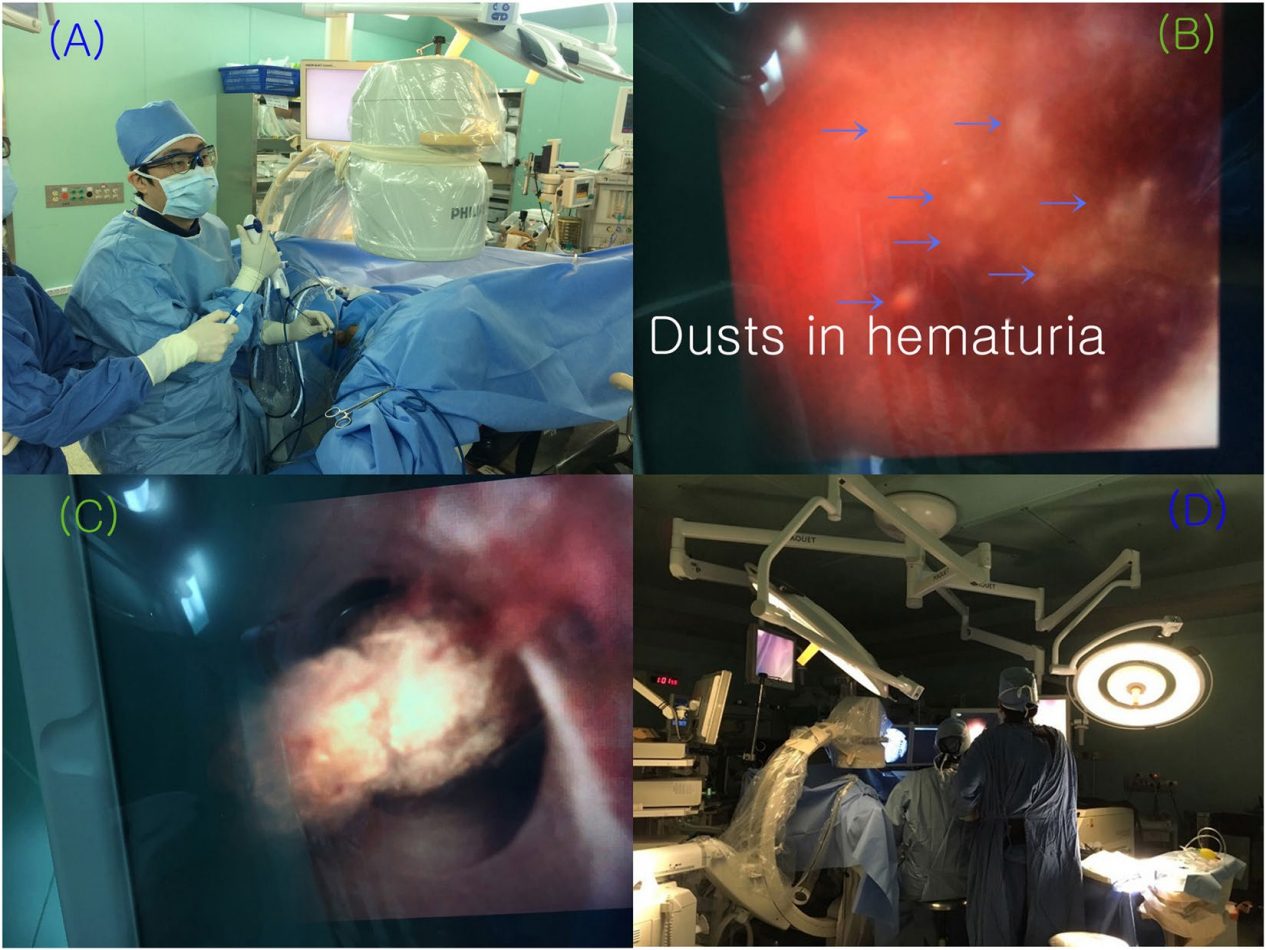
Various perspective of LITHOVUE. (**A**) Wrist and thumb movement during stone surgery using LITHOVUE. (**B**) Visualization of the monitor system during hematuria. The dusts do not clearly appear in the monitor. (**C**) Brightness of the monitor system. (**D**) Use of the astral lamp in a dark operating room.

The most important advantage of the device was excellent maneuverability. The deflection angles were perfectly preserved in 85.5%, and only 4 out of 62 cases (6.5%) showed less than 210 degrees. Surgeons did not worry about the deflection capacity of the scopes because they could use new ones in all cases. The lightness of the device lessened fatigue in surgeons’ shoulders and hands. Some surgeons gave poor evaluations on ‘wrist stiffness’ and ‘thumb fatigue’ because of the slippery surface of the plastic body during grasping of the scope. For surgeons with smaller hands the physical strain of a tight grip seemed to be higher than for those with larger hands (Fig. 1A). Scores may be affected by a surgeon’s position during surgery. However, overall, the scores were not different according to the surgeon’s position (3 surgeons in a sitting position and 2 surgeons in a standing position).

### Perceptions of the monitor system

Perception scores for the monitor system were generally good with a mean value of 2.5 (better to much better). Twenty-five cases (40.5%) scored it as excellent and 5 cases (8.1%) rated it as poor. LITHOVUE adopted complementary metal oxide semiconductor (CMOS) digital technology for better visualization, which can be compared to charge coupled devices (CCD). CMOS is cheaper and has more rapid data processing speed^11^. However, it’s more sensitive to the red color and the data analysis for balancing red color was problematic in cases with hematuria, which mean that the dusts do not clearly appear in the monitor. (Fig. 1B). Some surgeons felt that brightness was different between the central and the peripheral zones of the monitor system (Fig. 1C). However, this did not affect surgical outcomes. A final comment was the glistening surface of the monitor film (Fig. 1C). This performed better when surgeons turned off the light in the operating room and turn on the astral lamp light to avoid unnecessary reflected light (Fig. 1D).

### Perceptions of the irrigation system

The perception scores of the irrigation system were the worst among all parameters at 3.0 (no difference). Fifteen cases (24.2%) were assessed poorly and this was based on any situation requiring efficient ‘irrigation’ because the surgical procedure usually involved the dusting technique with some degree of hematuria. To guarantee a clear visual field, small diameters for baskets and laser fibers are recommended. Intermittent irrigation without devices in the working channel is also recommended. A dark operating room with the astral lamp on can be helpful.

### Limitations of the study

(1) This study does not provide the evidence about the sample size and power calculation because it is a single arm study. A larger series of cases would be helpful to evaluate perception scores more accurately because this study could have unavoidable errors considering discrepancies between surgeons and centers. (2) The surgeons in this study evaluated the scores comparing ones of their pre-existing flexible ureterorenoscopes (Flex-X2 from Karl Storz, Flex-X2S from Karl Storz, Flex-Xc from Karl Storz, URF-V2 from Olympus and Cobra from Wolf) in their institutions. However, the authors have tried to reduce these errors by unifying perioperative management, surgical techniques and medical devices. (3) The study design would have been much stronger if the LITHOVUE was directly compared with an or more than one reusable flexible ureteroscope. However, there are many different types of flexible ureteroscopes and the 1:1 matched comparison would have advantages and disadvantages as well. Therefore, the authors decided to include comparative parameters about maneuverability of the scopes, perceptions of the visual monitor system and the visibility during stone fragmentation procedures for relative evaluation. And the investigators planned to focus on the performance of the flexible ureterorenoscopes. (4) Although Fig. 1B showed that the monitor system did not show the dusts clearly, Boston Scientific recently updated the software of the monitor system. The present study could not show the updated image. (5) Finally, the authors think that these ‘comparative parameters’ in the Result section would show valuable information to the surgeons who consider using the disposable flexible ureterorenoscopes. Interestingly, the results showed the same tendency in all surgeons and this would imply that the disposable flexible ureterorenoscopes have definite advantages in the performance parameters.

## Conclusion

The LITHOVUE showed generally excellent maneuverability and good perception scores for the monitor and irrigation systems in Asia population. Strong points were maneuverability and the monitor system, while the weak point was predominantly related to situations requiring efficient irrigation. The weak point can be overcome by appropriate surgical methodology. No pre-stenting procedure and the longer operative time were significant predictors of poorly-preserved maximal deflection angles <270 postoperatively.
